# Immunogenetic Epidemiology of Multiple Sclerosis in 14 Continental Western European Countries

**DOI:** 10.29245/2578-3009/2021/2.1216

**Published:** 2021-05-18

**Authors:** Lisa M. James, Apostolos P. Georgopoulos

**Affiliations:** 1 The HLA Research Group, Brain Sciences Center, Department of Veterans Affairs Health Care System, Minneapolis, MN, 55417, USA; 2 Department of Neuroscience, University of Minnesota Medical School, Minneapolis, MN 55455, USA; 3 Department of Psychiatry, University of Minnesota Medical School, Minneapolis, MN 55455, USA; 4 Department of Neurology, University of Minnesota Medical School, Minneapolis, MN 55455, USA

**Keywords:** Multiple sclerosis, Human leukocyte antigen, Epidemiology, Immunity, Genetics

## Abstract

Human leukocyte antigen (HLA), a system involved in immune response to foreign antigens and in autoimmunity, has been strongly implicated in multiple sclerosis (MS). Prior research has shown that HLA DRB1*15:01 exerts the strongest susceptibility effect, although other HLA alleles have been implicated in both susceptibility to, and protection against, MS. Here we utilized an immunogenetic epidemiological approach to evaluate correlations between the population frequencies of 127 HLA Class I and II alleles and the population prevalence of MS in 14 Continental Western European countries to identify an HLA profile for MS. The results of these analyses, which largely corroborated prior findings and revealed several novel and highly robust HLA associations with MS, revealed a larger number of protective HLA alleles than susceptibility alleles, particularly for HLA Class I. Given the role of HLA in pathogen elimination and autoimmunity, these findings point to a contributory role of exposure to pathogens in the absence of protective HLA in underlying the inflammation and autoimmunity associated with MS.

## Introduction

Multiple sclerosis (MS), a chronic autoimmune inflammatory disease of the central nervous system, is the most common neurological disorder in young adults. According to recent estimates^1^, 2.8 million people globally are living with MS, and the global prevalence is increasing^1,2^. Pathologically, MS is characterized by multifocal demyelinating lesions, axonal loss, atrophy, and perivascular inflammatory infiltrates^3^. Symptoms of MS vary tremendously and can include problems with mobility, hand function, vision, fatigue, cognition, bowel/bladder function, sensory, spasticity, pain, depression, and tremor/coordination^4^. Although the course is variable, the vast majority of MS patients have a relapsing-remitting form of the disease^5^. A specific cause has not been identified; however, there is strong evidence that exposure to pathogens such as the Epstein-Barr virus may influence risk of MS, suggesting a potential role of viral influence in addition to the more traditional views of MS as an autoimmune disorder or as an inflammatory disease that may give way to neurodegeneration at advanced stages^6,7^. Additional lifestyle and environmental factors such as smoking, sun exposure/vitamin D, and adolescent obesity also appear to play a role in MS susceptibility^8,9^. Finally, there is considerable geographic variation in MS prevalence^6^. It has been well-established that a latitudinal gradient exists for MS^10^, leading researchers to speculate that environmental factors that vary with latitude such as ultraviolet radiation or vitamin D are associated with the increasing disease prevalence at higher latitudes; however, anomalies in the general latitudinal gradient, particular for indigenous populations, suggest the influence of additional factors that influence population susceptibility to MS^6,10^.

A system that varies by population, is involved with pathogen elimination (e.g., Epstein-Barr virus) and autoimmunity, and has been strongly implicated in MS is Human Leukocyte Antigen (HLA). HLA genes code for cell-surface proteins that facilitate immune system responses aimed at elimination of foreign antigens. HLA Class I molecules (HLA-A, -B, -C) present intracellular antigen peptides to CD8+ cytotoxic T cells to signal destruction of infected cells. HLA Class II molecules (HLA-DR, DQ, and DP genes) present endocytosed extracellular antigen peptides to CD4+ T cells to promote B-cell mediated antibody production and adaptive immunity. Thus, HLA confers protection via pathogen elimination; consequently, HLA has evolved in concert with pathogen evolution^11,12^ and has been shown to vary by population^13,14^, presumably reflecting differences in pathogen exposure. In fact, HLA is the most highly polymorphic region of the human genome, thereby maximizing species resistance to foreign antigens. However, a breakdown in this process – for instance, an inability of the immune system to distinguish self from non-self antigens – can result in autoimmunity^15^. Strong evidence suggests that MS is an autoimmune disease directed against myelin or other CNS antigens^16^. Class II HLA genes account for 10.5% of the genetic variance underlying risk for MS with HLA DRB1*15:01 exerting the strongest effect, although additional alleles, varying across populations, have been inconsistently implicated in MS^17–21^. With regard to immunogenetic protection, the most consistent protective effects have been reported for Class I HLA-A*02:01, although in European Populations DRB1*14:01 has also been shown to be highly protective^17^. Moreover, environmental factors that have been implicated in MS susceptibility have been shown to interact with HLA to influence risk^8,9^.

As noted elsewhere^17^, a limitation of many of the HLA associations identified in MS, with the exception of DRB1*15:01, is that they have been detected through imputation rather than direct HLA sequencing. While more time- and cost-effective than direct sequencing, the accuracy of HLA imputation for certain alleles (e.g., DRB1) has been reported to be as low as 30%^22^. Conversely, due to the highly polymorphic nature of HLA, direct sequencing of large enough samples to identify HLA-disease associations is often prohibitive. Here we take an alternative immunogenetic epidemiology approach that takes advantage of the population heterogeneity of HLA and permits identification of a high-resolution population level HLA profile with regard to disease prevalence. Using this approach, we^23–26^ and others^13^ have identified HLA alleles that are negatively associated with disease prevalence (i.e., protective alleles) as well as HLA alleles that are positively associated with disease prevalence (i.e., susceptibility alleles). In light of HLA’s involvement in pathogen elimination and autoimmunity, we presume that protective alleles facilitate pathogen elimination and that susceptibility alleles promote autoimmunity. Here, we extend that line of research to identify an HLA profile for MS in Continental Western Europe using population-level immunogenetic data and MS prevalences estimated from the 2016 Global Burden of Disease study, the most comprehensive global study of disease morbidity, mortality, and associated risk factors. Though studies at the individual or cohort level permit more direct investigation of MS pathogenesis and factors that affect MS disease course and treatment response^27–29^, immunogenetic epidemiological approaches such as ours are beneficial for understanding immunogenetic contributions to population-level disease variability.

## Materials and Methods

### Prevalence of MS

The population prevalence of MS was computed for each of the following 14 countries in Continental Western Europe: Austria, Belgium, Denmark, Finland, France, Germany, Greece, Italy, Netherlands, Portugal, Norway, Spain, Sweden, and Switzerland. Specifically, the total number of people with MS in each of the 14 Continental Western European countries as determined by the Global Burden of Disease study^2^, which utilizes rigorous, standardized approaches to estimate country-specific data from multiple sources, was divided by the total population of each country in 2016 (Population Reference Bureau)^30^ and expressed as a percentage. We have previously shown that life expectancy for these countries are virtually identical; therefore, life expectancy was not included in the current analyses.

### HLA

The frequencies of all reported HLA alleles of classical genes of Class I (A, B, C) and Class II (DPB1, DQB1, DRB1) for each of the 14 Continental Western European countries were retrieved from the website allelefrequencies.net (Estimation of Global Allele Frequencies^31,32^) on October 20, 2020. There was a total of 2746 entries of alleles from the 14 Continental Western European countries, comprising 844 distinct alleles. Of those, 127 alleles occurred in 9 or more countries and were used in further analyses. The distribution of those alleles to the HLA classes and their genes is given in Table 1.

## Data analysis

HLA profiles for MS were derived as described previously for Parkinson’s disease and dementia^26^. Briefly, the prevalence of MS in a country was computed as the fraction of total country population and was expressed as a percentage. MS prevalences were natural-log transformed^23–26^ and the Pearson correlation coefficient, r, between MS prevalence and the population frequency of each one of the 127 HLA alleles above calculated and Fisher z–transformed^33^ to normalize its distribution:

$${\text{r}}^{\prime }=\text{atanh}(\text{r})$$

The MS HLA profile consisted of 127 values of $r^{\prime }$ The effects of HLA Class and gene (within a class) on $r^{\prime }$ were evaluated using a univariate analysis of variance (ANOVA). Finally, differences in the proportions of the counts of negative and positive $r^{\prime }$ were evaluated using the Wald H0 statistic for comparing proportions of independent samples. Statistical analyses were performed using the IBM–SPSS package (IBM SPSS Statistics for Windows, Version 26.0, 64–bit edition. Armonk, NY: IBM Corp; 2019) and Intel FORTRAN (Microsoft Visual Studio Community Version 16.8.3; Intel FORTRAN Compiler 2021).

## Results

**Figure 1.**
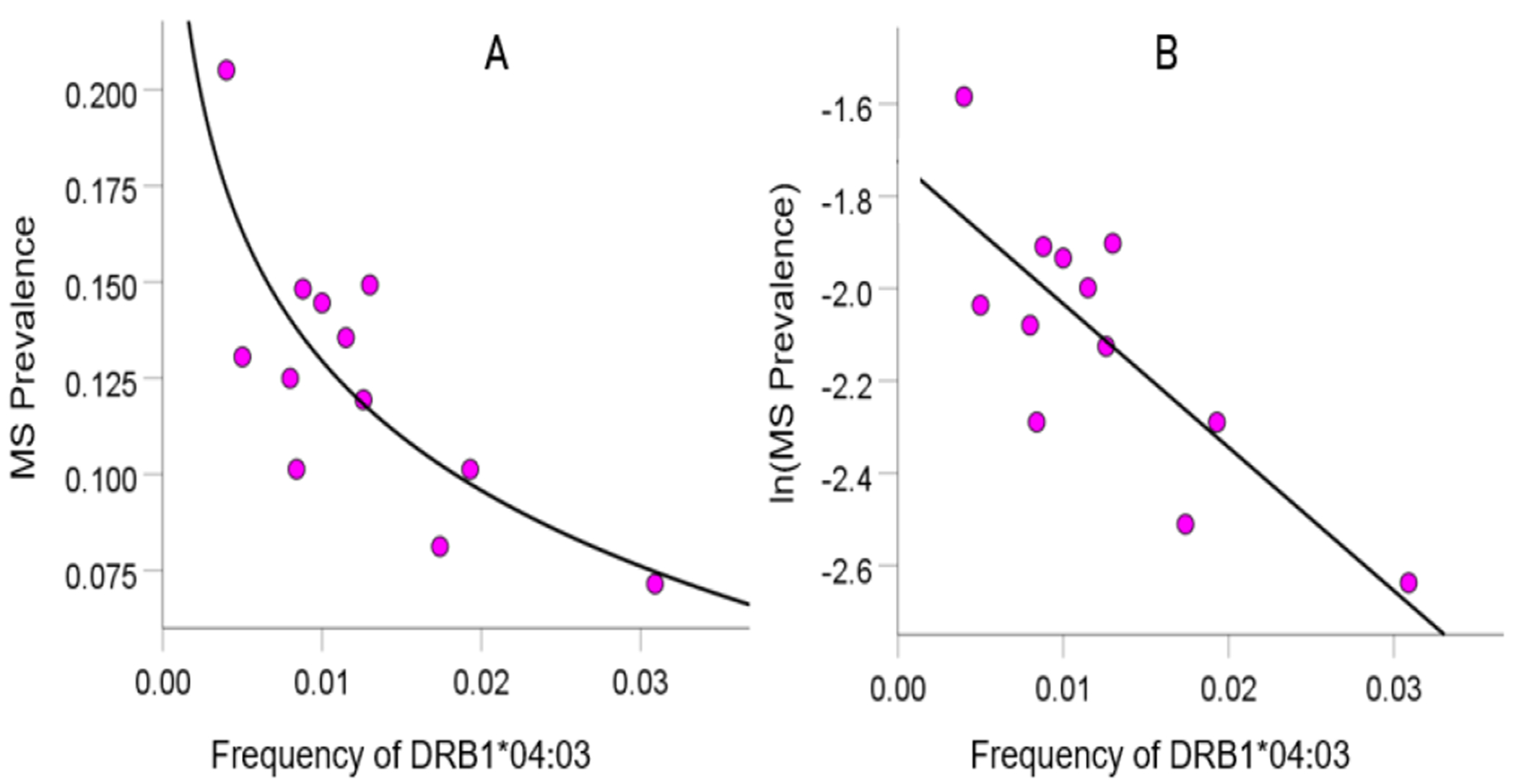
Example from a presumed protective HLA allele (DRB1*04:03) for MS. A, MS prevalence (%) for 12 CWE countries is plotted against the corresponding frequency of the DRB1*04:03 allele; the fitted line is a logarithmic fit. B, the same data are plotted using the natural-log transformed MS prevalence against the same allele frequency; the line is a linear fit (P = 0.002).

**Figure 2.**
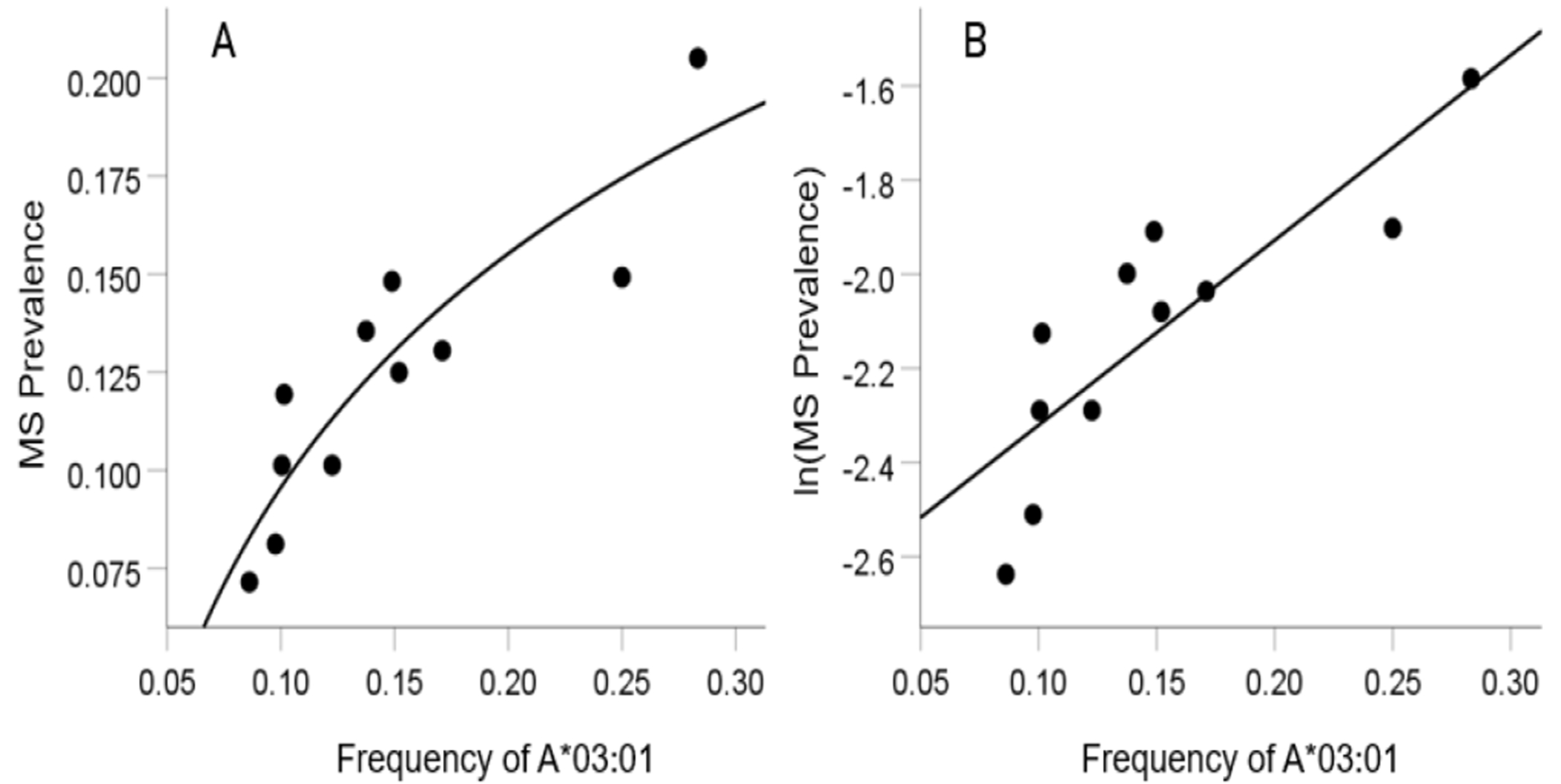
Example from a presumed susceptibility HLA allele (A*03:01) for MS. A, the prevalence for 11 CWE countries is plotted against the corresponding frequency of the A*03:01 allele (logarithmic fit); B, the same data are plotted using the natural-log transformed MS prevalence against the same allele frequency; the line is a linear fit (P = 0.001).

As mentioned above, the MS HLA profile consists of correlations between allele frequency and disease prevalence, suitably Fisher z-transformed (Equation 1) to normalize their distribution for further analyses. We showed previously^24^ that dementia prevalence varies in an exponential fashion with allele frequency, such that the logarithm of disease prevalence is a linear function of allele frequency. We found the same relation here between MS prevalence and HLA allele frequency. Two examples are illustrated in Figures 1 and 2, namely for a presumed MS protective allele (DRB1*04:03) and a susceptibility allele (A*03:01) (Figures 1 and 2, respectively).

## HLA-MS profile

**Figure 3.**
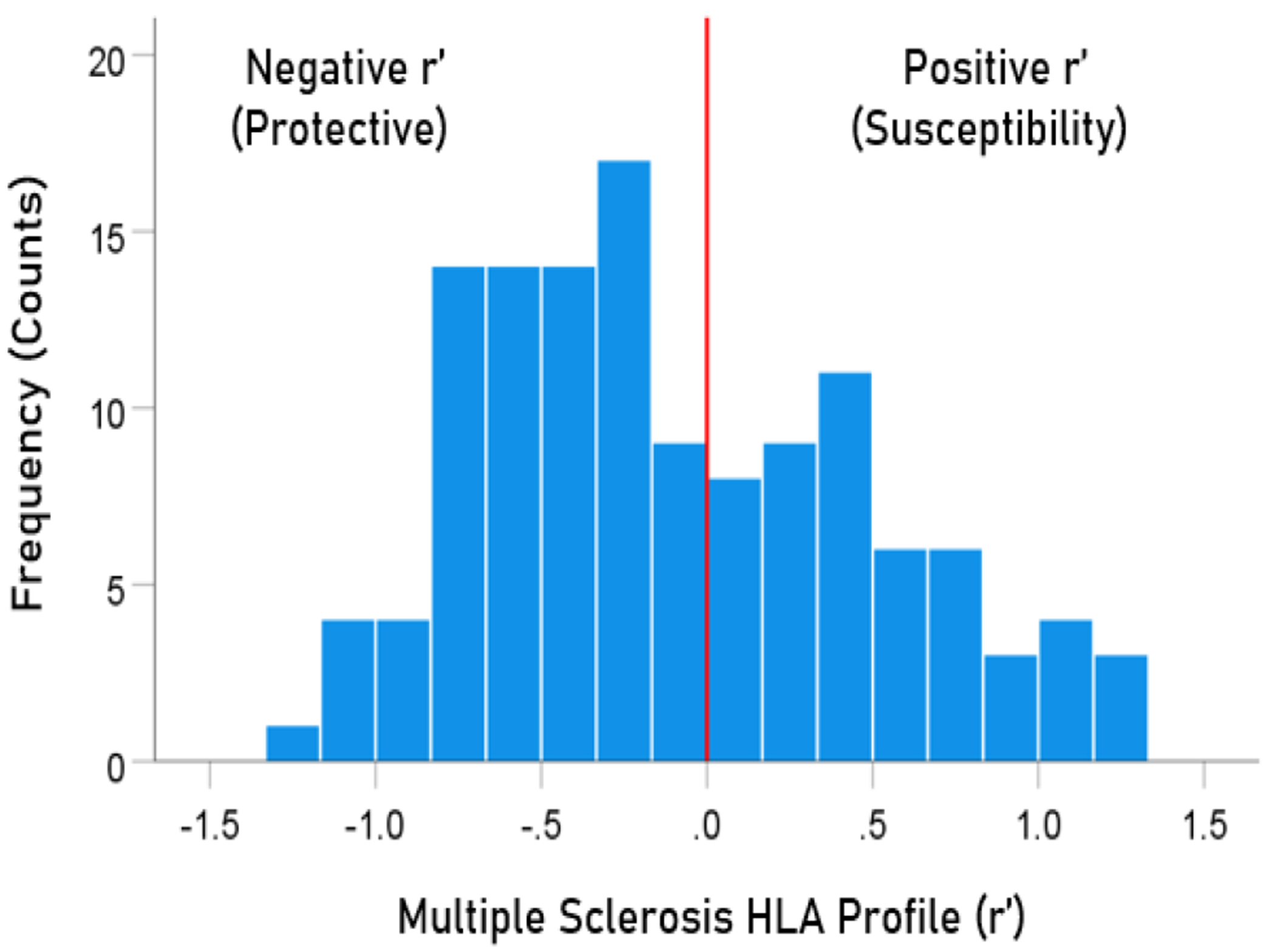
Frequency distribution of MS HLA profile (N = 127).

The frequency distribution of alleles in the MS HLA profile (Table 2) is shown in Figure 3. There were 77/127 (60.6%) negative (protective) alleles and 50/127 (39.4%) positive (susceptibility) alleles. These percentages differed significantly from the null hypothesis of 50% (P = 0.017, two-sided one-sample binomial test; z = 2.396).

**Figure 4.**
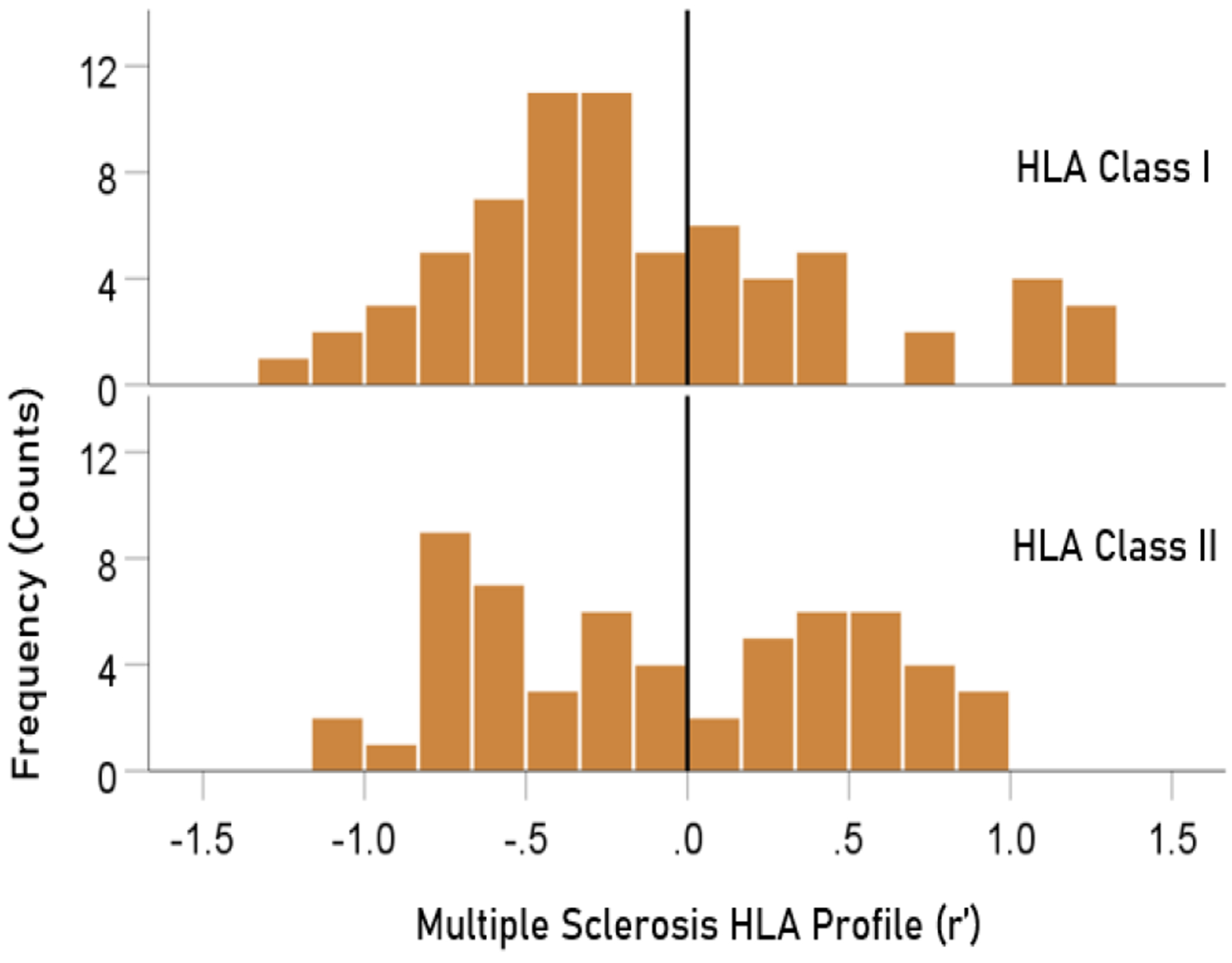
HLA Class distributions of MS HLA profile . N = 69 alleles for Class I and 58 alleles for Class II.

The distributions of $r^{\prime }$ for Class I and II are shown in Figure 4. There were 69/127 (54.3%) $r^{\prime }$ in Class I and 58/127 (46.7%) in Class II; these percentages did not differ significantly (P = 0.329, two-sided one-sample binomial test; z = 0.976). For Class I, there were 45/69 (65.2%) negative (protective) and 24/69 (34.8%) positive (susceptibility) values, respectively; these percentages differed significantly (P = 0.011, two-sided one-sample binomial test; z = 2.528). In contrast, for Class II, there were 26/58 (44.8%) negative and 32/58 (55.2%) positive values, respectively; these percentages did not differ significantly (P = 0.431, two-sided one-sample binomial test; z = 0.788).

## Analysis of strength of $r^{\prime }$

There were no statistically significant differences in the strength of $r^{\prime }$ $\left(\left|r^{\prime }\right|\right)$ between the two HLA classes or among HLA genes (within Classes) (P > 0.3 for all comparisons, ANOVA).

## Discussion

Here we used an immunogenetic epidemiological approach across 14 countries in Continental Western Europe to identify a population-level HLA profile consisting of protective and susceptibility alleles for MS. The results, which generally corroborate and extend existing literature regarding HLA susceptibility and protection in European populations, demonstrated a larger number of protective HLA alleles than susceptibility alleles, particularly for Class I HLA. These findings are discussed within the context of the role of HLA in pathogen elimination and autoimmunity.

The evolutionary role of Class II HLA is host protection from foreign pathogens via facilitation of antibody production and adaptive immunity; however, Class II HLA are also strongly associated with autoimmune diseases, including MS, in which autoantibodies target self-antigens^15^. While several non-mutually exclusive mechanisms have been proposed to explain the association of Class II HLA with autoimmune disorders^15^, the specific mechanisms that increase risk of MS among carriers of certain Class II HLA alleles remain inconclusive^21,34^. Nonetheless, it is well-established that Class II HLA, and DRB1*15:01 in particular, has a strong genetic predisposing effect on MS^17–21^. Consistent with the existing literature, a strong correlation between the population frequency of DRB1*15:01 and the population prevalence of MS was found here ($r^{\prime }$ = 0.823). In addition, several additional HLA susceptibility alleles were identified, many of which exhibited even stronger associations with the population prevalence of MS including DRB1*01:01, DRB1*04:01, DRB1*04:08. In contrast, several Class II HLA alleles were shown to be highly protective against MS at the population level in the present study. Of the Class II HLA alleles, DRB1*14:01 has most consistently been associated with protection from MS in European populations^17^. Other Class II alleles such as DRB1*11 have been shown to be protective against MS in Brazilian^35^ and Canadian^36^ populations. Here, DRB1*14:01 as well as all of the DRB1*11 alleles were protective against MS. Some studies have reported protective effects for HLA-DRB1*01 and DRB1*10^37^; however, it appears these alleles are protective only in the presence of HLA-DRB1*15 and do not exert independent protective effects where as DRB1*14 and DRB1*11 exert dominant independent protection^36^. Consistent with those findings, we did not find protective effects for DRB1*01:01 or DRB1*10 in the present study. We did, however, identify several other alleles that appear to provide population-level protection against MS. Of the 26 Class II HLA protective effects observed here, the strongest protective effects were found for DRB1*04:03, DQB1*05:02, and DPB1*14:01.

Similar to the findings for Class II, both protective and susceptibility effects were observed for Class I HLA with regard to the population prevalence of MS. In light of the role of Class I HLA in pathogen elimination via signaling destruction of infected cells, the Class I protective effects observed here are presumably related to efficient pathogen elimination. Of the 45 protective Class I alleles, the strongest effects were observed for HLA-A*11:01, HLA- A*32:01, HLA-B*18:01, HLA-B*35:08, HLA-B*38:01, HLA-B*40:01, HLA-B*49:01, and HLA-B*51:01. Although several of these allele associations with MS appear to be novel, prior research has found protective effects for HLA-B*35:08^38^. Several studies including some in European cohorts using direct HLA genotyping have reported protective effects of A*02^17^; here, HLA-A*02 was not protective and was only weakly associated with MS prevalence. Although Class I HLA alleles were significantly more likely to be protective (i.e., negatively associated with MS prevalence), there were several Class I HLA alleles that were positively associated with MS. Of those, the strongest effects were observed for HLA-A*03:01, HLA-A*37:01, HLA-B*07:02, HLA-B*15:01, HLA-B*37:01, HLA-C*07:02, Some of these alleles (e.g., HLA-A*03:01 and HLA-B*07:02)^39^ have previously been shown to be associated with MS risk though the findings have been inconsistent particularly with regard to whether the associations with disease are independent or accounted for by linkage disequilibrium with other alleles (e.g., DRB1*15:01)^17^. Others appear to be newly identified susceptibility alleles. Several viral and non-viral mechanisms that have been proposed to explain the role of Class I HLA in autoimmunity^15^; although, as with Class II HLA involvement in MS, the specific mechanisms linking Class I HLA to MS risk are inconclusive^21,39^.

The strength of the current population-level study rests in the ability to evaluate the association of a large number of high-resolution Class I and Class II HLA alleles with MS prevalence across countries. In contrast to imputation methods which have been shown to have questionable accuracy and are often limited to 2-digit resolution^17^, HLA genotype data used in this study was determined by direct sequencing at high-resolution (4-digit). Given the highly polymorphic nature of HLA, low-resolution genotyping may mask important protein-level differences in disease associations. For instance, here we found that DRB*04:01 and DRB*04:04 and DRB*04:08 are positively associated with MS prevalence whereas DRB1*04:02, DRB1*04:03, DRB1*04:05, and DRB1*04:07 are protective (i.e., negatively associated). Similarly, DRB1*15:01 is highly associated with MS risk in the present study (and elsewhere) but DRB1*15:02 was found to be highly protective. Allele group (i.e., 2-digit) resolution obscures these important distinctions. In addition, this across countries immunogenetic epidemiological approach permits identification of alleles that are broadly relevant to MS within this geographic region.

The present findings must also be considered within the context of study limitations. First, these population-level findings lay the groundwork for their validation at the individual level to confirm the HLA-disease associations observed here. Second, it is unclear if the current findings regarding HLA-disease associations observed in these 14 Continental Western European countries extend to other regions. Third, previous research has identified haplotypes that are associated with MS, the most widely reported being HLA-DRB1*15:01–DQA1*01:02–HLA-DQB1*06:02. We did not evaluate the effect of haplotypes on MS risks and instead focused on the association of individual alleles on MS prevalence. Of note, recent research has demonstrated that the most widely recognized haplotype effects are indeed driven by a single allele - HLA-DRB1*15:01^19^. Finally, environmental factors have been shown to interact with HLA to influence MS risk^8,9^. An evaluation of interactions between 127 HLA alleles and environmental factors is beyond the scope of this paper. Future studies examining the influence of HLA and environmental factors represents an important area of research to help discern factors that contribute to MS that could potentially be mitigated to reduce MS risk.

## Conclusion

It is widely accepted that MS is likely a result of genetic and environmental interactions. Here we have corroborated several prior findings and identified novel HLA genes that are associated with MS. Given the role of HLA in pathogen elimination and autoimmunity, these findings point to a contributory role of exposure to pathogens in the absence of protective HLA in underlying the inflammation and autoimmunity associated with MS.

## Figures and Tables

**Table 1:** Distribution of 127 HLA alleles analyzed to Class and Genes.

	Class I (N = 69)			Class II (N = 58)		
Gene	A	B	C	DPB1	DQB1	DRB1
Count	20	36	13	15	14	29

**Table 2:** HLA profile of MS. The signed z-transformed correlation coefficient ($r^{\prime }$) between 127 HLA alleles and ln (MS) prevalence. N denotes the number of CWE countries from which $r^{\prime }$ was calculated.

	Allele	Class	N	$r^{\prime }$(MS)
1	A*01:01	I	11	−0.158
2	A*02:01	I	11	0.185
3	A*02:05	I	9	−0.251
**4**	A*03:01	**I**	**11**	**1.240**
**5**	A*11:01	**I**	**11**	**−0.941**
6	A*23:01	I	11	−0.731
7	A*24:02	I	11	0.324
8	A*25:01	I	12	0.299
9	A*26:01	I	11	−0.637
10	A*29:01	I	11	0.077
11	A*29:02	I	11	−0.355
12	A*30:01	I	11	−0.071
13	A*30:02	I	12	−0.356
**14**	A*31:01	**I**	**9**	**1.216**
**15**	A*32:01	**I**	**12**	**−0.968**
16	A*33:01	I	10	−0.292
17	A*33:03	I	9	−0.548
18	A*36:01	I	10	−0.335
19	A*68:01	I	11	0.078
20	A*68:02	I	10	0.094
**21**	B*07:02	**I**	**10**	**1.027**
22	B*08:01	I	12	0.344
23	B*13:02	I	11	0.133
24	B*14:01	I	11	−0.332
25	B*14:02	I	10	−0.372
**26**	B*15:01	**I**	**10**	**1.184**
27	B*15:17	I	9	−0.081
28	B*15:18	I	9	−0.563
**29**	B*18:01	**I**	**12**	**−1.056**
30	B*27:02	I	10	0.032
31	B*27:05	I	12	0.776
32	B*35:01	I	11	−0.068
33	B*35:02	I	9	−0.265
34	B*35:03	I	9	−0.603
**35**	B*35:08	**I**	**9**	**−0.831**
**36**	B*37:01	**I**	**10**	**1.059**
**37**	B*38:01	**I**	**9**	**−0.899**
38	B*39:01	I	11	−0.294
39	B*39:06	I	9	−0.329
**40**	B*40:01	**I**	**12**	**1.130**
41	B*40:02	I	12	0.437
42	B*41:01	I	11	−0.282
43	B*41:02	I	10	−0.656
44	B*44:02	I	12	0.065
45	B*44:03	I	12	−0.396
46	B*44:05	I	9	−0.752
47	B*45:01	I	10	−0.316
48	B*47:01	I	11	−0.214
**49**	B*49:01	**I**	**11**	**−1.008**
50	B*50:01	I	10	−0.420
**51**	B*51:01	**I**	**10**	**−1.260**
52	B*52:01	I	10	−0.485
53	B*55:01	I	11	0.351
54	B*56:01	I	9	0.459
55	B*57:01	I	12	−0.391
56	B*58:01	I	9	−0.383
57	C*01:02	I	9	0.381
58	C*03:03	I	9	0.709
59	C*04:01	I	9	−0.501
60	C*05:01	I	9	0.266
61	C*06:02	I	9	−0.221
62	C*07:01	I	9	−0.187
**63**	C*07:02	**I**	**9**	**1.160**
64	C*07:04	I	9	−0.404
65	C*12:02	I	9	−0.635
66	C*12:03	I	9	−0.739
67	C*14:02	I	9	−0.780
68	C*15:02	I	9	−0.491
69	C*16:01	I	9	−0.024
70	DPB1*01:01	II	11	0.526
71	DPB1*02:01	II	11	−0.714
72	DPB1*02:02	II	10	−0.264
73	DPB1*03:01	II	11	0.229
74	DPB1*04:01	II	11	0.562
75	DPB1*04:02	II	11	−0.271
76	DPB1*05:01	II	11	0.405
77	DPB1*06:01	II	10	0.247
78	DPB1*09:01	II	9	−0.106
79	DPB1*10:01	II	10	−0.715
80	DPB1*11:01	II	9	−0.111
81	DPB1*13:01	II	10	−0.649
**82**	DPB1*14:01	**II**	**11**	**−1.068**
83	DPB1*17:01	II	9	−0.201
84	DPB1*19:01	II	11	0.245
85	DQB1*02:01	II	12	0.459
86	DQB1*02:02	II	11	−0.327
87	DQB1*03:01	II	13	−0.693
88	DQB1*03:02	II	13	0.748
89	DQB1*03:03	II	13	0.812
90	DQB1*04:02	II	13	0.615
91	DQB1*05:01	II	13	0.267
**92**	DQB1*05:02	**II**	**10**	**−0.844**
93	DQB1*05:03	II	12	−0.682
94	DQB1*06:01	II	11	−0.181
95	DQB1*06:02	II	14	0.629
96	DQB1*06:03	II	13	0.177
97	DQB1*06:04	II	12	0.503
98	DQB1*06:09	II	9	−0.707
**99**	DRB1*01:01	**II**	**14**	**0.838**
100	DRB1*01:02	II	11	−0.693
101	DRB1*01:03	II	11	−0.550
102	DRB1*03:01	II	13	−0.041
**103**	DRB1*04:01	**II**	**13**	**0.983**
104	DRB1*04:02	II	11	−0.692
**105**	DRB1*04:03	**II**	**12**	**−1.079**
106	DRB1*04:04	II	13	0.425
107	DRB1*04:05	II	9	−0.166
108	DRB1*04:07	II	12	−0.216
**109**	DRB1*04:08	**II**	**9**	**0.842**
110	DRB1*07:01	II	12	−0.581
111	DRB1*08:01	II	13	0.623
112	DRB1*08:03	II	11	0.042
113	DRB1*09:01	II	12	0.668
114	DRB1*10:01	II	14	0.035
115	DRB1*11:01	II	14	−0.337
116	DRB1*11:02	II	12	−0.533
117	DRB1*11:03	II	12	−0.619
118	DRB1*11:04	II	12	−0.611
119	DRB1*12:01	II	13	0.478
120	DRB1*13:01	II	14	0.423
121	DRB1*13:02	II	14	0.470
122	DRB1*13:03	II	10	−0.494
123	DRB1*13:05	II	10	−0.651
124	DRB1*14:01	II	14	−0.371
125	DRB1*15:01	II	13	0.823
126	DRB1*15:02	II	10	−0.771
127	DRB1*16:01	II	10	−0.669

**Note.** The strongest associations are in bold.
